# Assessment of executive functions using a 3D-video game in children and adolescents with ADHD

**DOI:** 10.3389/fpsyt.2024.1407703

**Published:** 2024-08-22

**Authors:** Nagahide Takahashi, Tomihiro Ono, Yuka Omori, Misuzu Iizumi, Hidekazu Kato, Shinichi Kasuno, Barry Persing, Kenji J. Tsuchiya

**Affiliations:** ^1^ Department of Child and Adolescent Psychiatry, Nagoya University Hospital, Nagoya, Japan; ^2^ Research Center for Child Mental Development, Hamamatsu University School of Medicine, Hamamatsu, Japan; ^3^ United Graduate School of Child Development, Osaka University, Kanazawa University, Hamamatsu University School of Medicine, Chiba University, and University of Fukui, Suita, Japan; ^4^ Department of Research and Development, Almaprism Inc., Kyoto, Japan

**Keywords:** ADHD, video game, executive function, ecological validity, cognitive ability

## Abstract

**Objective:**

Executive functions are important factors that affect the well-being of children with ADHD. Therefore, inclusion of a convenient assessment of executive dysfunction in diagnosis and treatment of ADHD patients is warranted. However, executive dysfunction assessment presently relies on lab-based neuropsychological tests and symptom rating scales. The present study examined the potential of a 3-D action puzzle video game to reflect ecologically valid executive functioning in pediatric ADHD patients.

**Methods:**

Participant gameplay metrics were compared to both their Cambridge Neuropsychological Test Automated Battery (CANTAB) and Conners 3 Parent Form’s executive functioning subscale scores. Participants consisted of 33 ADHD and non-ADHD patients aged 8-21.

**Results:**

Metrics from gameplay were associated with distinct CANTAB test scores, and a composite score from gameplay was significantly correlated with executive dysfunction from Conners 3.

**Conclusion:**

For children with ADHD, cognitive domains related to executive function and overall real-life executive functioning appear to both be measurable via video games. It may be possible to develop individualized behavioral therapy based on the quantitative data obtained from the video game used in this study.

## 1 Introduction

Attention-deficit hyperactivity disorder (ADHD) is an often-lifelong neuro-developmental disorder that impacts children and adults worldwide, with estimates consistently placing prevalence at around 6-10% in children (1, 2). Symptoms of ADHD put patients at higher risk of educational failure, lower productivity, loss of employment, substance abuse, traffic accidents, obesity, and reliance on social services (3), with early detection and intervention/treatment being a key factor in improving long-term prognosis and longitudinal outcomes (4). Though therapeutic drugs are effective in improving symptoms (5), specifically concerning attention, vigilance, and hyperactivity, their effectiveness is limited against higher-order cognitive and executive functions such as planning and scheduling tasks (6). Though ADHD in the pediatric population receives much attention, recent reports suggest long-lasting persistence and impacts of ADHD into adulthood as well, with serious and debilitating clinical, educational, professional, and economic ramifications (3, 4, 7).

Executive functions have been variously described as the ability to regulate one’s own actions in a logical, sequential manner to achieve goals set by the individual and engage in novel problem solving (6, 8–11). Executive functions mobilize other simpler cognitive abilities including working memory, switching, and inhibitory control (9), which in turn serve as building blocks for more complicated cognitive abilities like planning (12). There is varying consensus on the degree to which executive function can be fractionated into independent module-like domains, but it is generally agreed upon that executive functioning involves a complex interplay of various domains and that the particular profile of deficit (i.e. which components a patient may be impaired in) has important implications for their behavioral and clinical outcomes (6, 8–10). As many as 50% of pediatric ADHD patients exhibit deficits in one or more executive function components (10), and executive functions strongly influence the quality of life (QOL) and mental health of ADHD patients (13–16). The type of executive dysfunction a patient has may also strongly influence what psychosocial treatments may be effective or appropriate (6, 8, 17, 18). As such, it is important to assess executive (dys)function in a way that is relevant to ADHD pathology and psychosocial interventions. A common approach is to employ carefully curated and refined digital tasks drawing upon traditionally administered neuropsychological tests (19–23) and real-life-relevant rating scales and interviews as recommended (24), but the two do not always correlate (11, 25, 26), confounding diagnostic and treatment efforts. A possible explanation for this discrepancy is that neuropsychological tests and real-life rating scales reflect related but different types or levels of thinking. The well-defined structural problem-solving of neuropsychological tests likely is fitted to reflect aptitude in algorithmic thinking, whereas the ill-defined and iterative problem-solving required in real-life situations draws more upon awareness and reflective thinking (27–29).

Neuropsychological tests draw upon a rich history of non-invasive examinations of brain damage patients to provide precise population-normed estimates of certain defined cognitive functions (9, 30). Neuropsychological batteries such as the CANTAB cognitive assessment battery (Cambridge Neuropsychological Test Automated Battery - Cambridge Cognition Ltd., Cambridge, UK) (31) may be used to fractionate executive function into components such as planning, working memory, problem solving, and inhibition. This is in contrast to symptom rating scales such as the Conners 3 Rating Scale Parents’ Form (32) for ADHD, which may be used to rate to what extent the patient can perform when faced with problems and situations relevant to their day-to-day functioning. Clinical practice guidelines note that neuropsychological assessments provide information helpful in learning about a child’s strengths and weaknesses such that appropriate psychosocial interventions may be crafted (24).However, clinicians are also aware that even well-established neuropsychological tests do not track with self- or parent-rated real-life executive dysfunction (9, 30, 33–35) and test features or testing protocols may mask some deficits that are pertinent in real-life situations (25, 36, 37).

Rating scales, on the other hand, involve the patient, their parent/guardian, or their teacher responding to a series of preset items. The responses are then appropriately converted and tallied, to yield a score that tells the clinician to what extent the patient experiences symptoms and problems in their day-to-day situations compared to their peers. Rating scales are often useful in gauging the presence of symptoms in multiple settings (home, school, work, *etc.*) as recommended by clinical guidelines (38). However, rating scales by themselves have limitations when it comes to the evidence-based assessment and treatment of ADHD, particularly of executive dysfunction. It can be difficult for the clinician to pinpoint an appropriate psychosocial intervention from rating scales alone: a rating score informs the clinician of how the rater perceives struggles relative to standards set by the patient’s environment (*e.g.* how well their peers are doing) and rating scales alone do not allow for clinicians to parse specific deficits, since the problems arise under the influence of a myriad of environmental factors that cannot be controlled or eliminated as they can be in laboratory tests (39). Additionally, rating scales are imperfect, in diagnostic accuracy (40), rater dependence (6, 41), and susceptibility to bias (42), such that guidelines for practice warn against overdependence on rating scales (24).

As such, both neuropsychological tests and rating scales come with their own limitations. Traditional neuropsychological tests offer an array of precise results but scores and performance on said tests do not always correlate with a patient’s symptoms and concerns (25, 36) because the controlled testing environment and limited tasks do not mimic real-life situations and problems (33, 35). Rating scales offer direct, relevant tallies of a patient’s symptoms and concerns, but risk having rater subjectivity and environmental influences baked-in. And even if both were thoroughly administered, executive functioning is a multi-faceted higher-order cognitive process and may not be fully captured in the combination of fractionated, isolated tasks (neuropsychological tests) and recall-dependent verbalizations (rating scales).

In other words, specifically in the context of executive function testing for ADHD patients, popular methods leave much to be desired in the realm of “ecological validity”—the relevance of a measure to a patient’s performance in real-life contexts.

We acknowledge that the commonly used meaning of “ecological validity” has shifted since its inception (43) and in the present paper include two related but distinct concepts in referring to “ecological validity”: the inclusion of test features or conditions that mimic those seen in real-life situations (*i.e.*, “does this test mimic conditions similar to real-life situations that the patient encounters?”), and relevance to real-world problems and clinical symptoms (*i.e.*, “is this test relevant to/informative of a patient’s actual real-life struggles?”). A measurement method that can reflect cognitive abilities (especially those components relevant to executive function, such as planning, working memory, *etc.*) while also being more relevant to real-life scenarios and situations could provide a new approach to assessing executive functioning in ADHD patients. As one possible solution to the above need, we considered the use of a video game as an assessment of executive function in pediatric ADHD patients.

Video games have diversified in form and factor since their initial introductions to the consumer market and now serve as an umbrella term for everything from casual screen-tapping mobile games to heavily strategic and complex operations played on dedicated equipment (44). In parallel, efforts to “gamify” otherwise boring tasks have boomed, with varying degrees of success (45). In the present paper, we focus on video games as relevant to complex cognition: requiring deliberative, cerebral engagement and the orchestration of higher-order cognitive processes to achieve success (46). Additionally, we focus on multimedia applications that have rich interactivity built-in with entertainment in mind (45), providing moderate and novel challenges that are important for testing the mental capabilities of the player (47).

Our approach was motivated by the following two questions.

First, can video games reflect cognitive abilities, especially those that are core components or fractionations of executive function? Video games have been reported to reflect traditionally tested cognitive abilities such as visual search (48), fluid intelligence (46, 49–52) as well as wayfinding (53–55) despite the form factor being quite different from that of traditional neuropsychological tests.

Second, can video games mimic real-life scenarios and situations more than traditional neuropsychological battery tests? Commercially available video games tend to share features such as input modalities with high degrees of freedom, visual distractors and cues present on-screen, and a notable lack of step-by-step instructions save a brief introductory sequence. As these features parallel some key features of real-life situations absent in traditional neuropsychological testing (35, 36), we hypothesized that examining executive function performance in the context of a video game environment would yield ecologically valid, clinically useful information about the executive dysfunction of pediatric ADHD patients.

### 1.1 Objectives

In this paper we examined results from a custom-built action puzzle game capable of outputting five metrics: *Maximum Difficulty Solved* (planning ability), *Task-Appropriate Coordinate Repeat* (set shifting), *Score Attack Deliberation* (inhibition), *Score Attack Verbosity* (working memory), and *Score Attack Record* (strategic thinking). We hypothesized based on how the game balance and data collection algorithms were structured that the game and its associated metrics could enable behavioral phenotyping of in-game actions while also remaining relevant to real-life ADHD symptoms, specifically executive functioning.

Specifically, we aimed to answer the following questions:

- What associations do metrics from the action 3-D video game exhibit with respect to traditional neuropsychological assessment scores (as measured through CANTAB)?
- What associations do metrics from the action 3-D video game exhibit with parental ratings of day-to-day executive function (as measured through Conners 3)?

## 2 Materials and methods

### 2.1 Participants and procedure

A total of N = 33 individuals (24 boys, 73%, ages 8-21) from Central Japan participated in this study.

Participants were recruited from outpatients at Nagoya University Hospital’s Department of Child and Adolescent Psychiatry as well as from volunteers living in the cities of Nagoya and Hamamatsu, and their surroundings. We recruited for children who could participate along with a parent or guardian, and required participants to not have diagnoses for epilepsy, intellectual disability, or video game addiction. Potential participants were also ineligible if for whatever reason maneuvering the video game with a commercial game controller would be difficult. The protocol was set up to be for Japanese-speaking participants.

Once registered, the participant first completed three computerized cognitive assessment tasks (Stop-Signal Task, Spatial Working Memory, and One-Touch Stockings from the CANTAB cognitive assessment battery) on a computer tablet with touchscreen controls. Then, the participant played the 3-D action puzzle video game on a desktop computer using a commercial video game controller. A medical professional was present to monitor and guide the process for the whole time. Meanwhile, the participant’s parent/guardian in a separate room responded to an extended questionnaire about various psycho-pathological and behavioral characteristics of the participant.

The study protocol was approved by the Nagoya University Hospital Ethics Committee (Ref. 2023-0028, “Study of measuring executive functions related to ADHD using video games”) and was conducted in accordance with the principles outlined in the Declaration of Helsinki. Written informed consent was obtained from participants over 16 years old and their caregivers. Written informed assent was obtained from participants under 15 years old.

### 2.2 Assessment via cognitive battery

We obtained traditional measures of cognitive ability through three tests in the CANTAB Connect Research battery. Specifically, we administered the following three tests relevant to executive function.

1. The Stop-Signal Task, or SST, measures impulsivity with visual cues that sometimes conflict with auditory cues. Participants are given two input buttons on the left and right sides of the screen, and are instructed to respond as quickly as possible to an on-screen cue telling them which button to press. After a practice cycle, the participants are then instructed to listen for an auditory cue that sometimes plays in tandem with the visual cue and inhibit their response if they hear the auditory cue, but otherwise respond as quickly as possible if there is no auditory cue (56, 57).
2. The Spatial Working Memory test, or SWM, assesses working memory accuracy and strategy with a 2-dimensional visual task. The participant is presented with a series of rounds in which one of the scattered boxes on the screen contains a hidden token. Participants select the boxes one by one to search for the token. When a token is found, the participant makes progress in the task, and repeats the process for several more rounds. They are instructed in the beginning that a box that contains a token will not contain a token in future rounds, so the number of times in which they revisit a box that previously contained a token is taken to be a proxy of working memory error (57, 58).
3. The One-Touch Stockings test, or OTS, which assesses planning ability with a sequence prediction task similar to the Tower of Hanoi puzzle. Participants are presented with three columns containing colored balls, analogous to balls placed in a vertical stocking. Balls can be moved to other columns, but only if there are no other balls on top of it. Participants are then expected to figure out the minimum sequence of moves required to take the stockings from one configuration of balls to another, with successive problems requiring longer and longer planning sequences (57, 59).

### 2.3 Assessment via video game

**Figure 1:**
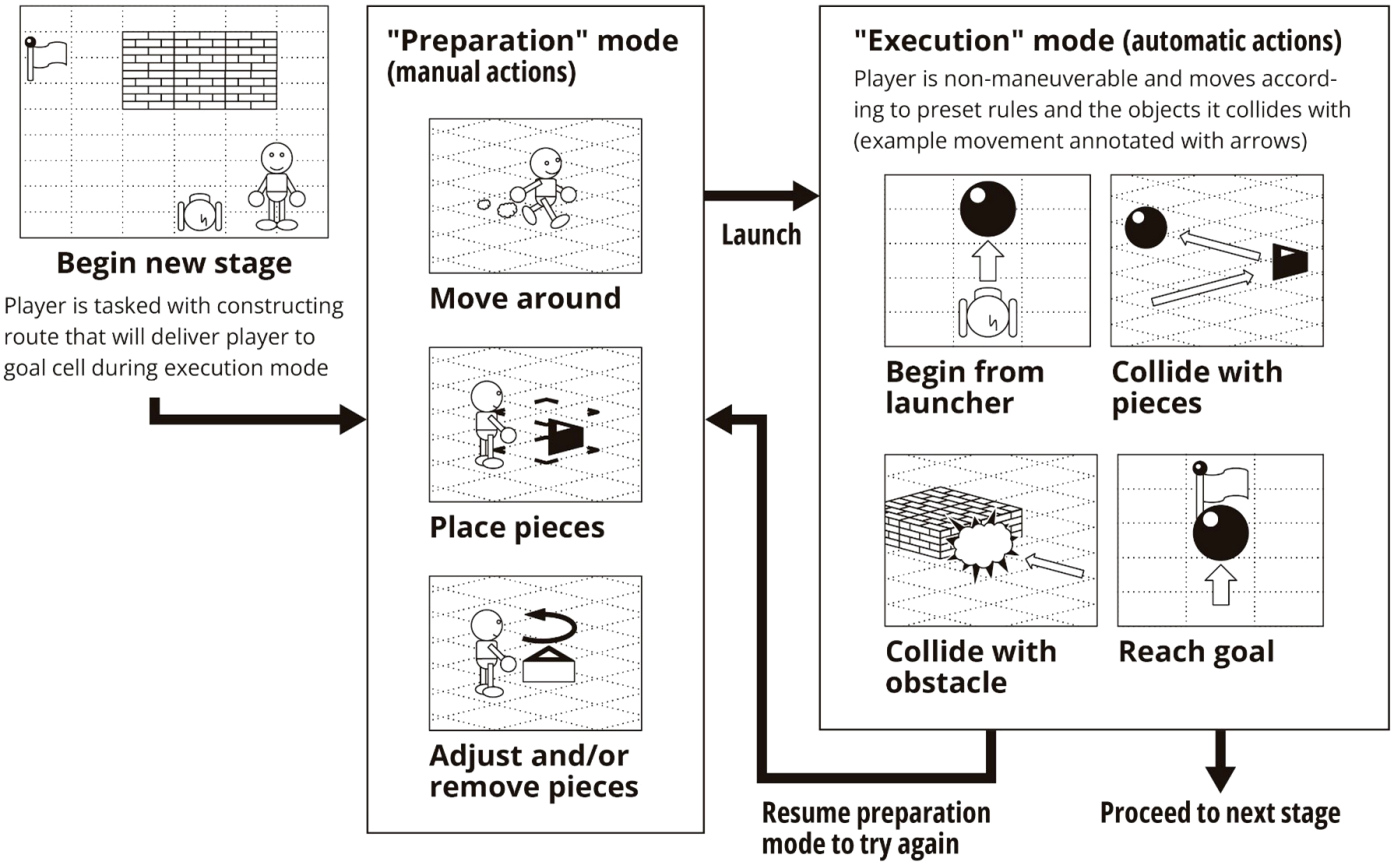
A graphical summary of key actions and events in relation to the manual "preparation" mode and automatic “execution” mode. The player is tasked with placing and adjusting pieces such that the resulting route can deliver an automatically moving player to the goal cell during execution mode.

The video game we utilized in this study to explore ecologically valid assessment of executive function in pediatric ADHD patients was developed by Almaprism Inc. (Kyoto, Japan) in collaboration with the authors and takes the form of a 3-D action puzzle game. The game program was run on a Microsoft Windows computer, and the player provided inputs via a Microsoft Xbox controller with a wired connection. The game consisted of a number of “stages” (tasks) of varying difficulty in sequence, with one measurement session lasting 45 minutes in terms of play-time. For a summary of key gameplay elements, see **Figure 1**.

The main gameplay experience consisted of two mutually exclusive modes between which the player can switch at any time. Broadly, “Preparation” was for constructing the solution, and “Execution” was for trying that solution out and processing the result (success or failure, and observing if the solution worked as intended). The objective in every stage was to construct an automatic path from the launch point coordinate to the goal coordinate. In order to meet the objectives, the player needed to imagine how automatic movement would proceed based on how the stage looked with the “pieces” that have been placed and plan out adjustments or piece additions as necessary. Pieces are objects that can redirect the automatic path of the player.

During “Preparation”, the player could move an avatar around freely in the virtual 3-D environment and adjust their point of view by rotating the virtual camera, and place pieces of their choosing near their avatar, as well as adjust previously placed pieces (*e.g.* adjust orientation, remove). Since the player in this phase does not yet know the exact outcome of the pieces they placed, additions and changes made in this phase could be likened to the “initial planning” as proposed by Davies (60): premeditated, hierarchical, and orderly.

During “Execution”, the player lost the ability to freely move around or place/adjust pieces and began automatic movement from the “launch point” coordinate. While in this automatic movement mode, the player could only change their point of view, and the avatar would continue movement until obstructed by an in-game object (either previously placed pieces or preexisting stage objects) at which point the avatar would respond automatically based on preset rules. If the avatar successfully reached the goal coordinate the player won the stage. If the avatar collided with a non-piece surface the attempt was counted as a failure and the player resumed preparation mode. The player does not have the ability to make changes to the setup during execution, but (on failure) the preparation mode that immediately follows can be likened more to Davies’ “concurrent planning”: opportunistic, *ad hoc*, and reactive (60). As such, the player’s behavior prior to their first execution likely merits distinction from player behavior after the first execution.

The first 5 minutes of gameplay were devoted to a tutorial sequence in which the player was introduced to the basic rules and inputs of the game. Then, the player progressed through the “Puzzle Sequence”—a preset stage sequence of graduated difficulty levels with increasing constraints for 30-35 minutes (the player tried a given stage repeatedly until they were able to clear it). Finally, the last 10-15 minutes were devoted to a repeating “Score Attack” stage in which there were no obstructors and the player was tasked with passing through preset “score” tiles as many times as possible in one execution to get the highest score that they can.

At specific timings, the game program submitted quantitative data about the player’s gameplay (both inputs and results) to an external secured server. The following five video game metrics were calculated per-player.

#### 2.3.1 Maximum difficulty solved (planning ability)

The highest difficulty stage that the player was able to solve in the Puzzle Sequence, regardless of how long they took or how many attempts they took, was recorded as a measure of their ability to solve logically complex tasks, analogous to performance measurement in planning tasks like the One-Touch Stockings task (57, 59).

#### 2.3.2 Task-appropriate coordinate repeat (set shifting)

In the Puzzle Sequence, complex piece placement tended to make the task more difficult (here, complexity was operationalized by counting the number of times a player’s execution route visited the same spatial coordinate), so the incentives favored relatively minimally complex, manageable piece placement. On the other hand, in the Score Attack the player generally benefited from visiting the same coordinate multiple times (i.e., higher coordinate repeat count). The difference between “the Score Attack z-score of the log of the maximum complexity the player constructs” and “the highest per-Puzzle-Sequence-stage z-score of the log of the maximum complexity the player constructs” (where z-scores were relative to other players’ metrics, pooled per stage, since different stages had different baseline complexities that are required) was recorded as a measure of the player’s ability to alter their input patterns according to what was called for by the task at hand.

#### 2.3.3 Score attack deliberation (inhibitory control)

In the Score Attack, since there were no obstructors and no “minimum” hurdles to clear logic-wise, how long the player wanted to deliberate and place pieces until their first execution was entirely up to the player. The log of the number of seconds the player took in the Score Attack until their first execution was recorded as a measure of the player’s tendency to deliberate. This measurement, though on a longer several-minute timespan than the sub-second measurements typically obtained in the Stop Signal Task (57), was analogous to traditional inhibitory control measurements. It should alos be noted that since this is inhibition prior to the first execution, this metric corresponds to initial planning (60) and does not include concurrent planning for the Score Attack task.

#### 2.3.4 Score attack verbosity (working memory)

In the Score Attack the incentives were in favor of verbose piece placement, but placing more pieces imposed a cognitive load specifically on working memory because for each new piece placement the player had another factor to consider when planning out the next piece placement. The log of the maximum number of pieces attempted in one Execution (regardless of whether the pieces were used or successful) during Score Attack was recorded as a measure of the player’s working memory capacity.

#### 2.3.5 Score attack record (strategic thinking)

In the puzzle sequence, when the player cleared a given task they were immediately given a new task, but in the Score Attack, the player had to re-examine their existing solution and make modifications to it to aim for a higher score. The log of the highest score achieved during their Score Attack was recorded as a measure of the player’s ability to fluidly strategize, as a measure of performance in ill-defined problem solving (61) in direct contrast to the well-defined problem solving performance measured in Maximum Difficulty Solved.

### 2.4 Rating scale/questionnaire contents

An experienced psychologist with a Ph.D. administered the extended rating scale/questionnaire administered to the participant’s parent/guardian, and through it we obtained demographic, pathophysiological, and behavioral information regarding the participant. Items relating to ADHD characteristics were obtained through the Conners 3 Parents’ Form. Items relating to Autism Spectrum Disorder (ASD), a common co-occurring condition in ADHD patients, were obtained through the Social Responsiveness Scale 2 (SRS-2). Additionally, the questionnaire contained items pertaining to demographic information, such as age, sex, accommodations at school, and digital device usage.

### 2.5 Statistical analysis

Our primary goal was to examine the extent to which participants’ cognitive abilities and symptoms as traditionally measured were reflected in their video game metrics. First, we examined the distributions of the video game metrics and performed a log-transform for metrics with excessive skew. Next, we took the video game metrics and examined their correlations with traditional metrics (neuropsychological batteries and rating scales) to check if the video game metrics were reflecting behavior or abilities that we expected based on their definitions. Then, we examined if the video game metrics, if any, exhibited significant association with real-life executive functioning as reported by the Conners 3 Parents’ Form.

Lastly, we conducted hierarchical linear regression to examine whether the associations between video game metrics and real-life executive functioning persisted after the addition of demographic covariates. Numerous demographic variables have been reported to affect participant video game performance, for example sex (62–65), age (52, 66), and habitual or prior video game exposure (67). As such, we chose to first examine the direct relationship between the video game metric and the traditional metric in isolation, and then add background factors as covariates to see if the relation holds.

We performed all computation of metrics and statistical modeling using R v. 4.1.3 (68), RStudio (69), and the tidyverse package (70).

## 3 Results

### 3.1 Sample characteristics

The sample’s descriptive statistics are summarized in **Table 1**. A total of thirty-three participants between eight and twenty-one years old took part in the study, with the average age being 13.45 years old (+- 2.96 years, s.d.) and 73% being male. Twenty-two of the participants had received an ADHD diagnosis by a board-certified child psychiatrist (N.T.) in the past (67% of total) of which twelve had received an ASD diagnosis (36% of total, 54% of ADHD participants), and twenty-one (twenty among ADHD participants) were on some psychiatric prescription at the time of participation (64% of total, 91% of ADHD participants). We did not have any non-ADHD participants with a prior ASD diagnosis. Age- and sex-normed t-scores for ADHD (Conners 3) and ASD (SRS-2) symptoms revealed that participants generally displayed more pathology than the general population, regardless of their official ADHD or ASD diagnosis status. Specifically, the average executive function t-score as measured by the Conners 3 Parents’ Form was 60.48 (+- 12.55, s.d.), indicating that the average participant showed a notable level of executive dysfunction in day-to-day situations according to their parent.

**Table 1:** Sample characteristics.

	n	%
Total	33	
Demographics		
Male	24	73
Clinical Information		
ADHD Diagnosis	22	67
ASD Diagnosis	12	36
Receives Medication	21	64
	Mean	SD
Demographics		
Age	13.45	2.96
Video Game Metrics		
Maximum Difficulty Solved, out of 11	9.03	1.91
Task-Appropriate Coordinate Repeat	-1.31	1.61
Score Attack Deliberation	5.37	0.59
Score Attack Verbosity	3.67	0.73
Score Attack Record	9.92	0.83
CANTAB Cognitive Battery		
One-Touch Stockings Score (OTS PSFC), out of 15	11.42	2.70
Stop-Signal Task Reaction Time (SST SSRT), ms	237.12	63.06
Spatial Working Memory Errors (SWM BE468)	7.15	6.99
Spatial Working Memory Strategy (SWM-S)	6.94	2.03
Conners 3 Parents’ Form		
Inattention (IN) t-score*	61.49	13.87
Hyperactivity/Impulsivity (HY) t-score*	55.50	13.77
Learning Problems (LP) t-score*	58.70	12.58
Executive Functioning (EF) t-score*	60.48	12.55
Aggression (AG) t-score*	53.63	10.24
Peer Relations (PR) t-score*	67.68	25.69
Social Responsiveness Scale (SRS-2)		
Total t-score*	61.58	16.46
Social Communication and Interaction (SCI) t-score*	59.88	15.97
Restricted Interests and Repetitive Behavior (RRB) t-score*	65.42	16.93

*Normed by age and sex according to technical manual values.

The five video game metrics (Maximum Difficulty Solved, Task-Appropriate Coordinate Repeat, Score Attack Deliberation, Score Attack Verbosity, Score Attack Record) were scaled to z-scores.

### 3.2 Comparing video game metrics and neuropsychological battery results

Concerning the comparison of video game metrics to traditional neuropsychological assessments, four of the five metrics were found to be associated with their expected analogues in the CANTAB cognitive assessment battery. Maximum Difficulty Solved and OTS Number Correct, a measure of planning ability, had a correlation of *r* = 0.366 (p < 0.05). Score Attack Deliberation and SST Response Time (log-transformed), a measure of inhibitory control, had a correlation of *r* = -0.509 (p < 0.01). Score Attack Verbosity and SWM Errors, a measure of working memory, had a correlation of *r* = -0.503 (p < 0.01). Score Attack Record and SWM Strategy, a measure of strategic thinking ability, had a correlation of *r* = -0.575 (p < 0.001).

Notably, Task-Appropriate Coordinate Repeat did not correlate with any of the available CANTAB assessment scores despite being associated with real-life executive dysfunction, and Score Attack Verbosity—which we expect reflects working memory—was additionally correlated with SWM Strategy (*r* = -0.556, p < 0.001), the measure of strategic actions within the working memory neuropsychological test.

### 3.3 Comparing video game metrics and rating scale results

The five video game metrics were individually compared with the age-normed, sex-normed Executive Functioning t-score of the Conners 3 Parent’s Form, to see if real-life executive functioning was reflected in the in-game metrics. A summary of selected correlations can be seen in **Table 2**; a full table of correlations between video game metrics, the Conners 3 executive functioning score, CANTAB test scores, and demographic variables can be found in the Supplement.

**Table 2:** Selected correlations between video game metrics, rating scale, and neuropsychological tests.

		Rating Scale	Neuropsychological Tests			
		Conners 3 EF (executive function)	OTS Number Correct (planning ability)	SST Response Time (inhibition)	SWM Errors (working memory)	SWM Strategy (strategic thinking)
Metrics from Gameplay	Maximum Difficulty Solved (planning ability)	-0.361*	0.366*	-0.139	-0.255	-0.225
	Task Appropriate Coordinate Repeat (set shifting)	-0.362*	0.248	-0.102	-0.013	-0.301
	Score Attack Deliberation (inhibition)	-0.157	0.021	-0.509**	-0.158	-0.045
	Score Attack Verbosity (working memory)	0.069	0.295	-0.075	-0.503**	-0.556***
	Score Attack Record (strategic thinking)	-0.226	0.346	-0.355	-0.307	-0.575***
	Composite Score (executive function)	-0.405*	0.349	-0.130	-0.148	-0.295

Pearson’s r shown (***p < 0.001, **p<0.01, *p<0.05).

Only two out of the five output metrics were significantly associated with real-life executive dysfunction: Maximum Difficulty Solved (*r* = -0.361**, p < 0.01) and Task-Appropriate Coordinate Repeat (*r* = -0.362**, p < 0.01). Score Attack Deliberation, Score Attack Verbosity, and Score Attack Record were not found to be significantly associated with real-life executive dysfunction.

Since real-life executive dysfunction was correlated to two distinct video game metrics (Maximum Difficulty Solved and Task Appropriate Coordinate Repeat) which in turn displayed internal correlation (*r* = 0.575, p < 0.001), we employed hierarchical linear regression to determine if one held more explanatory sway than the other in terms of real-life executive dysfunction. When both were added as explanatory variables for real-life executive dysfunction neither metric was dominant, and a composite game metric (consisting of Maximum Difficulty Solved and Task Appropriate Coordinate Repeat averaged together) exhibited greater explanatory power than either of the two metrics alone. Lastly, this composite game metric retained significance after the addition of covariates (age, sex, and gaming habit). A summary of the hierarchical linear regression process can be seen in **Table 3**. As this composite score contains two key facets of executive function (planning and set shifting), in subsequent analyses we will refer to this as the composite EF (executive function) score.

**Table 3:** Hierarchical linear regression of the composite executive function score.

	Dependent Variable: Conners 3 EF (z-score)				
	(1)	(2)	(3)	(4)	(5)
Maximum Difficulty Solved (z)	-0.453* (0.210)	–	-0.277 (0.263)	–	–
Task Appropriate Coordinate Repeat (z)	–	-0.463* (0.221)	-0.299 (0.270)	–	–
Composite EF Score (z) *(MDS+TACR)/2*	–	–	–	-0.575* (0.241)	-0.624* (0.264)
Age	–	–	–	–	-0.007 (0.080)
Sex (0 = male, 1 = female)	–	–	–	–	-0.211 (0.508)
Gaming Habit (0 = no, 1 = yes)	–	–	–	–	0.509 (0.519)
Constant	–	–	–	–	0.870 (1.210)
Observations	33	31	31	31	31
R^2^	0.130	0.131	0.164	0.164	0.200
Adjusted R^2^	0.102	0.101	0.104	0.135	0.077
Residual Standard Error	1.190 (df = 31)	1.213 (df = 29)	1.211 (df = 28)	1.190 (df = 28)	1.229 (df = 26)
F Statistic	4.641* (df = 1; 31)	4.366* (df = 1; 29)	2.747 (df = 2; 28)	5.687* (df = 1; 29)	1.624 (df = 4; 26)

Hierarchical linear regression leading to the composite EF score from game metrics. *p<0.05; **p<0.01; ***p<0.001.

### 3.4 Adverse events

In terms of adverse events and otherwise unexpected occurrences, one participant experienced motion sickness during gameplay and terminated their participation, resting for a bit before going home. One participant experienced a nosebleed before starting the game, apparently from excitement, but was otherwise able to complete the game without any issues after being treated for the nosebleed. For one participant there were technical difficulties around data collection and some of their data points were lost.

## 4 Discussion

The present study aimed to explore the feasibility of extracting metrics that each relate to cognitive constructs relevant to executive function as classically defined, while also performing an ecologically valid performance measurement of executive function via a video game interface. Concerning the former, five video game metrics extracted from separate points in the gameplay displayed distinct correlations with results from traditional neuropsychological test scores, suggesting that it is possible to measure higher-order, composite performance like executive functioning while at the same time extracting useful information about component cognitive abilities from separate parts of the process. Concerning the latter, the composite EF score (calculated from the average of two video game metrics) suggests promise, if only in the limited instance of executive function as relevant to ADHD pathology in a pediatric population. This is particularly notable considering that in the same sample of participants (aged 8-21) including ADHD patients, there was no directly observable correlation between traditional neuropsychological assessments and real-life executive functioning problems.

### 4.1 Behavioral phenotyping with video game metrics

We found that four of the five metrics obtained from various points in gameplay each distinctly correlated with scores from conceptually analogous traditional neuropsychological tests. Maximum Difficulty Solved was significantly correlated with the CANTAB OTS score (a measure of planning ability), indicating that the graduated difficulty levels in the action 3-D puzzle game overlapped with the modified digital Tower-of-London task in its cognitive demands. Score Attack Deliberation was significantly related to the CANTAB SST Response Time (log-transformed; a measure of inhibitory control), with the negative coefficient reflecting differing instructions: in the SST, patients are instructed to respond as quickly as possible but inhibit responses when appropriate; in Score Attack, it is left up to the player when they wish to try out their deliberated plan. Score Attack Verbosity exhibited a significant correlation with both SWM Errors (working memory) and SWM Strategy (strategic thinking in the working memory test). The negative correlation with SWM Errors suggests that patients with higher working memory fidelity and capacity make fewer errors and tend to use more pieces in Score Attack. Finally, Score Attack Record exhibited a negative correlation with SWM Strategy.

There are several video game-based tools reported to succeed in assessing cognitive functions of ADHD (71–75). However, most of them used the Continuous Performance Test (CPT) as a gold standard for assessing executive function. Sustained attention in CPT is undoubtedly important in executive functioning, but does not comprehensively reflect executive functioning. We are not aware of studies using video games to assess multiple domains of executive functions in ADHD. We believe that our video game assisted behavioral phenotyping has advantages over previously reported video games in its breadth of metrics, and can be useful for developing personalized behavioral therapy based on the results.

### 4.2 The composite EF score

The composite EF score, calculated from a simple average of two game metric z-scores—Maximum Difficulty Solved and Task-Appropriate Coordinate Repeat—displayed greater explanatory power than either of the component variables alone with respect to real-life executive functioning issues as measured by the Conners 3 Parents’ Form (See **Table 3**). Based on a priori definitions and comparisons with neuropsychological tests, we had hypothesized that the two metrics reflect planning ability and set shifting, respectively, though the set shifting metric relies on definition more than validation since our neuropsychological battery did not include a set shifting test. A composite score related to planning and set shifting correlating with real-life executive functioning is not surprising, considering that the two constructs (or analogous fractionations) are often named as key components of executive functioning (9, 76–78). However, at the same time, it ought to be noted that in-game metrics thought to correspond to working memory and inhibition (Score Attack Verbosity and Score Attack Deliberation, respectively) displayed no correlation with real-life executive functioning issues, despite these two constructs being considered as core executive function components (9, 76) and important factors to consider when examining the manifestation of executive dysfunction in ADHD pathology (79, 80).

If planning, set shifting, working memory, and inhibition are all considered important fractionations of executive function, why in our sample did in-game planning and set shifting correlate with executive dysfunction (from Conners 3) while in-game working memory and inhibition did not? Two things can be true at once: that inhibition and working memory are important parts of executive dysfunction, and yet that they may not be powerful predictors of executive dysfunction in ADHD pediatric patients, especially when considered alongside higher-order cognitive functions such as planning and set shifting. Though working memory and inhibition are powerful predictors of performance in tasks that explicitly rely on those abilities with far-reaching consequences on day-to-day functioning and outcomes (81–83), in real life not all tasks have the same cognitive demands and patients are offered the ability to exercise considerable flexibility in how and when to tackle tasks, unlike during neuropsychological tests (33, 35). Especially for working memory and inhibition, in real life comparatively straightforward compensatory measures may be available, and/or their environment may not directly stress those abilities much. Notes, reminders, and partial solutions can remind a student what they were about to do, social cues to stay quiet may help a student from blurting out, and so forth. However, deficits in planning and set shifting are more likely to be consequential for the pediatric ADHD population: not having enough time to complete schoolwork, missing out on social engagements, hyperfixating on hobbies to the detriment of their performance, etc. In other words, the fact that in-game planning and set shifting were significantly associated with real-life executive functioning could suggest that for this population, deficits in planning and set shifting are overarching in determining outcomes. It may also be that in-game planning and set shifting were closer to the goal-oriented reflective level of thinking with ramifications in day-to-day functioning, whereas in-game working memory and inhibition were closer to the algorithmic level of thinking that can be easily supplemented or circumvented in real life (27).

Alternatively, since inhibition was measured through the SAD which definitionally excludes any concurrent planning behavior (60), it may be that impulse control as relevant to initial planning is less relevant to day-to-day executive dysfunction.

### 4.3 Video games for ecologically valid measurement?

Lastly, though not the main focus of this paper, we note that none of the neuropsychological test metrics we employed from the CANTAB digital cognitive assessment battery (OTS accuracy/planning ability, SST response inhibition time log, SWM error count/working memory, and SWM strategy) were significantly correlated (p < 0.05) with real-life executive dysfunction as measured by the Conners 3 Parent Form, despite these tests measuring cognitive abilities and constructs that are thought to be relevant or essential to executive function (9). These results echo past reports (35, 36, 77, 84) of the divide between the focused cognitive ability measurement of neuropsychological tests and level of function in real-life situations. Yet, in the same sample of participants, two video game metrics (Maximum Difficulty Solved/planning ability and Task-Appropriate Coordinate Repeat/set shifting) were each significantly correlated with real-life executive dysfunction.

Most instruments for measuring executive functioning as relevant to real-life situations either (1) systematically ask for subjective ratings of real-life problems or (2) test patients with tasks that tap into cognitive constructs and components relevant to executive function. A performance-based test of executive functioning has been elusive, especially in a form that does not require human raters and retains interpretability via correlations with tried-and-true neuropsychological assessments. We believe the present study demonstrates video games may be able to fulfill this performance-based test of executive function role.

Neuropsychological tests, for as useful as they are in non-invasively detecting brain lesion patients (30) and quantifying narrowly defined, interpretable cognitive constructs (9), also come with constraints that limit the transferability of results and insights to real-life situations. Input modalities are limited, for example primarily utilizing multiple choice as the only response modality (50), distractors are eliminated (except where relevant to the purpose of the test), and the proctor or digital assessment system acts as an ancillary cortex freeing the patient of some higher-order cognitive tasks like goal selection and monitoring (35). Compounding the dissimilarity is the fact that targeted tests are often chained together with minimal or nonexistent feedback, which implicitly tests the patient’s ability to rapidly switch between entirely different tasks over a prolonged duration. To what extent such conditions mimic real-life situations likely depends on the patient, but the general mismatch between neuropsychological tests and real-life executive functioning suggests that present techniques are not optimal for testing executive functioning performance.

Video games, on the other hand, generally offer more input modalities and distractors compared to neuropsychological tests, and where guidance does exist it is only to introduce players to the game system or provide loose guidance and have the player attempt however many times as it takes to achieve the goal. In other words, the minimal guidance offered to players renders video games an “ill-defined problem” in the beginning (61), with the player defining the problem through iteration and creative approaches being a key design element. The ability to incorporate feedback and gradually construct better and better methods of approaching complex tasks has not been fully explored in the literature, partly because learning effects complicate the work of measuring and interpreting neuropsychological abilities and properties, but it is not difficult to imagine that testing environments that can draw out such an ability would have resemblance or relevance to real-life situations in which patients are allowed to struggle with and overcome complex tasks. Additionally, video games through their multitude of available actions and maneuvers often allow for multiple ways to overcome an obstacle (50). Multiple pathways—especially when some involve compensatory behaviors that circumvent certain cognitive demands—are uncommon in neuropsychological tests for the sake of interpretability, but many real-life situations (*e.g.* learning from a lecture) allow for compensatory circumvention of cognitive demands (*e.g.* memory), some encouraged (*e.g.* taking notes) and some perhaps less so (*e.g.* taking a photo of the blackboard). Permitting and measuring how a patient maneuvers an environment with multiple pathways and solutions may provide more direct measurements of how a patient is responding to treatments and interventions aimed at improving their general health and disability, as well as quality-of-life.

We stress, however, that video games are not replacements for traditional neuropsychological batteries or rating scales; a complex digital task that allows for data collection relevant to both real-life situations and traditionally defined cognitive constructs comes with its own caveats, principally that it would be difficult to predict how a patient may perform in the face of a much less complex demand or how non-player factors (*e.g.* school or home environment, medical history, parental involvement) would be relevant to a patient’s condition and prognosis. As with other methods, we consider video games to be a promising avenue of ecologically valid measurement, to be used where necessary in addition to the many assessment methods already available for tackling the heterogeneity of ADHD pathology.

### 4.4 Limitations and future research

Some strengths of the present study include the use of a pathologically heterogeneous population, in terms of ADHD symptom severity and comorbidities. On the other hand, we also note several limitations of the present study that ought to be addressed in future studies and analyses.

First, the present study investigated the relationship of video game behavior metrics to parent-rated real-life executive functioning problems and neuropsychological test performance metrics in a mostly-pediatric population aged 8 to 21 years old (of varying ADHD severity). While the sample is suited for the purpose of the study, we also cannot predict how analogous investigations may conclude in non-pediatric population or with children preschool-aged or younger.

Second, though we recruited a mix of diagnosed and non-diagnosed participants of varying ADHD symptom severity and co-occurring conditions, the sample size and geographic distribution limits the generalizability of the findings reported in the present study. The sample size prevented meaningful analysis of subgroups, such as between ADHD subtypes or ASD-comorbid patients. In future studies we may incorporate short quantitative checklists such as the Short Autism Spectrum Quotient to examine the extent of ASD symptom co-occurrence (85).

Third, the participants were not recruited randomly but rather from the outpatient clinic at participating institutions, which likely skewed the sex distribution: up through adolescence, boys are more likely to be referred for ADHD symptoms and problems (24). This in turn limited our ability to address possible sex differences in video game performance.

Fourth, the range of participant ages is relatively wide (8-21 years), which in combination with the sample size did not permit for in-depth analyses of age-related effects. Though results from neuropsychological tests (CANTAB) and rating scales (Conners 3) were age-and-sex standardized per protocol (31, 32), there is still the possibility that the video game measurements were affected by the age of the participants.

Lastly, the present study concerns a one-time 45-minute session measurement, and cannot address whether the measurement results are stable over repeated measurements. As executive function primarily concerns action regulation to achieve goals and engage in novel problem solving, it is expected that repeated exposure to the same problems would reduce novelty, and possibly sever the link between video game performance and executive function (86). However, some facets of performance in complex video games have been reported to be consistently related to intelligence metrics despite extended practice periods, suggesting that not all video game cognitive metrics are susceptible to automation (47).

## 5 Conclusion

The present study used a novel assessment modality—an action 3-D puzzle game, to measure executive function and constituent constructs in parallel—in conjunction with validated and widely accepted neuropsychological tests and pathology rating scales in pediatric and adolescent ADHD patients to explore the feasibility of ecologically valid executive function measurement in video games. The results suggest that video games—when designed for the purpose of measurement, with distinct interpretable moving parts—can offer ecologically valid performance measurements more relevant to real-life executive functioning than traditional neuropsychological tests, while retaining relevance to said tests through a number of metrics collected in parallel from elsewhere in the gameplay. Utilizing design features of video games—the presence of distractors, higher degrees of freedom in input, the embracing of iteration, feedback, and learning—may offer new approaches to performance testing and measurement in clinical settings, especially concerning higher-order and complex cognitive functions that were previously difficult to quantify in an ecologically valid manner. The results from the present study are promising, but warrants further examination in future studies, including a replication of the present findings using an independent sample. We hope that continued development of performance-based executive function testing via video games will allow clinicians to tackle the heterogeneity of ADHD pathology with an even more multifaceted toolkit.

## Data Availability

The raw data supporting the conclusions of this article will be made available by the authors, without undue reservation.
