# Supplementary material for: Assessment of executive functions using a 3D-video game in children and adolescents with ADHD
Source: Front Psychiatry. 2024 Aug 22;15:1407703. doi: 10.3389/fpsyt.2024.1407703 (PMC11374763; doi:10.3389/fpsyt.2024.1407703)
Supplement: Supplementary file 1 [file Table1.docx]

**Supplement: Full Table of Correlations between Video Game Metrics and Traditional Metrics**

|  |  | Metrics from Gameplay | | | | | | Rating Scale (RS) | Neuropsychological Tests | | | | Demographic Variables | | | |
| --- | --- | --- | --- | --- | --- | --- | --- | --- | --- | --- | --- | --- | --- | --- | --- | --- |
|  |  | Maximum Difficulty Solved (planning ability) | Task Appropriate Coordinate Repeat (set shifting) | Score Attack Deliberation (inhibition) | Score Attack Verbosity (working memory) | Score Attack Record (strategic thinking) | Composite Score (executive function) | Conners 3 EF | OTS Number Correct (planning ability) | SST Response Time (inhibition) | SWM Errors (working memory) | SWM Strategy (strategic thinking) | Age | Sex (0 = male, 1 = female) | Gaming Habit (0 = no, 1 = yes) |  |
| Metrics from gameplay | Maximum Difficulty Solved (planning ability) | -- | 0.575*** | -0.105 | 0.505** | 0.543** | 0.891*** | -0.361* | 0.366* | -0.139 | -0.255 | -0.225 | 0.329 | -0.299 | 0.263 |  |
|  | Task Appropriate Coordinate Repeat (set shifting) | 0.575*** | -- | 0.068 | 0.041 | 0.713*** | 0.884*** | -0.362* | 0.248 | -0.102 | -0.013 | -0.301 | 0.103 | 0.150 | 0.048 |  |
|  | Score Attack Deliberation (inhibition) | -0.105 | 0.068 | -- | -0.229 | 0.131 | -0.022 | -0.157 | 0.021 | -0.509** | -0.158 | -0.045 | 0.300 | 0.292 | 0.036 |  |
|  | Score Attack Verbosity (working memory) | 0.505** | 0.041 | -0.229 | -- | 0.509** | 0.312 | 0.069 | 0.295 | -0.075 | -0.503** | -0.556*** | 0.085 | -0.553** | 0.516** |  |
|  | Score Attack Record (strategic thinking) | 0.543** | 0.713*** | 0.131 | 0.509** | -- | 0.706*** | -0.226 | 0.346 | -0.355 | -0.307 | -0.575*** | 0.179 | -0.080 | 0.207 |  |
|  | Composite Score (executive function) | 0.891*** | 0.884*** | -0.022 | 0.312 | 0.706*** | -- | -0.405* | 0.349 | -0.130 | -0.148 | -0.295 | 0.242 | -0.091 | 0.179 |  |
| RS | Conners 3 EF | -0.361* | -0.362* | -0.157 | 0.069 | -0.226 | -0.405* | -- | -0.303 | 0.317 | 0.007 | -0.171 | -0.172 | -0.051 | 0.116 |  |
| Neuropsychological Tests | OTS Number Correct (planning ability) | 0.366* | 0.248 | 0.021 | 0.295 | 0.346 | 0.349 | -0.303 | -- | -0.232 | -0.553*** | -0.348* | 0.061 | -0.123 | 0.046 |  |
|  | SST Response Time (inhibition) | -0.139 | -0.102 | -0.509** | -0.075 | -0.355 | -0.130 | 0.317 | -0.232 | -- | 0.113 | 0.204 | -0.594*** | -0.110 | 0.009 |  |
|  | SWM Errors (working memory) | -0.255 | -0.013 | -0.158 | -0.503** | -0.307 | -0.148 | 0.007 | -0.553*** | 0.113 | -- | 0.615*** | 0.087 | 0.224 | -0.254 |  |
|  | SWM Strategy (strategic thinking) | -0.225 | -0.301 | -0.045 | -0.556*** | -0.575*** | -0.295 | -0.171 | -0.348* | 0.204 | 0.615*** | -- | -0.073 | 0.257 | -0.223 |  |
| Demographic Variables | Age | 0.329 | 0.103 | 0.300 | 0.085 | 0.179 | 0242 | -0.172 | 0.061 | -0.594*** | 0.087 | -0.073 | -- | -0.049 | -0.115 |  |
|  | Sex (0 = male, 1 = female) | -0.299 | 0.150 | 0.292 | -0.553** | -0.080 | -0.091 | -0.051 | -0.123 | -0.110 | 0.224 | 0.257 | -0.049 | -- | -0.083 |  |
|  | Gaming Habit (0 = no, 1 = yes) | 0.263 | 0.048 | 0.036 | 0.516** | 0.207 | 0.179 | 0.116 | 0.046 | 0.009 | -0.254 | -0.223 | -0.115 | -0.083 | -- |  |

*Pearson’s r shown (***p < 0.001, **p<0.01, *p<0.05)*
